# Supplementary material for: Spatial biology using single-cell mass spectrometry imaging and integrated microscopy
Source: Nat Commun. 2025 Oct 15;16:9129. doi: 10.1038/s41467-025-64603-8 (PMC12528677; doi:10.1038/s41467-025-64603-8)
Supplement: Supplementary file 1 — Supplementary Information [file 41467_2025_64603_MOESM1_ESM.pdf]

Supplementary Information for

Spatial biology using single-cell mass spectrometry imaging and integrated microscopy

Alexander Potthoff [1], Jan Schwenzfeier [1], Marcel Niehaus [2], Sebastian Bessler [1], Emily Hoffmann [3], Oliver Soehnlein [4], Jens Höhndorf [2], Klaus Dreisewerd [1], Jens Soltwisch [1]

[1]Institute of Hygiene, University of Münster, Münster, Germany

[2]Bruker Daltonics GmbH & Co. KG, Bremen, Germany

[3]Clinic of Radiology, University of Münster, Münster, Germany

[4]Institute of Experimental Pathology, University of Münster, Germany

*correspondence addressed to: jenssol@uni-muenster.de

Contents:

Supplementary Figure 1:	Co-registration of external fluorescence image to t-MALDI-2 MSI across the different sample systems
Supplementary Figure 2:	Exemplary mass spectra of naïve and processed mouse brain tissue sections
Supplementary Figure 3:	Comparison of lipid signal intensities from murine cerebellum acquired with t-MALDI-MSI and reported values from bulk analysis
Supplementary Figure 4:	Murine 4T1 tumor t-MALDI-2 MSI overview.
Supplementary Figure 5:	Neighborhood analysis and lipid profile of neutrophils in context of their tissue microenvironment
Supplementary Figure 6:	Homogenous coating of samples with MALDI-matrix by resublimation.
Supplementary Note 1:	Determining the fidelity of co-registration between external FM and MSI
Supplementary Note 2:	Annotation of lipids in murine cerebellum and comparison to the literature
Supplementary Table 1:	List of lipid annotations from murine cerebellum samples
Supplementary Table 2:	Comparison of lipid annotations from untreated and stained murine cerebellum samples
Supplementary Table 3:	List of lipid annotations in 4T1-tumor samples
Supplementary Table 4:	Table of resources
Supplementary Table 5:	Light microscopy reporting table

Supplementary Figures

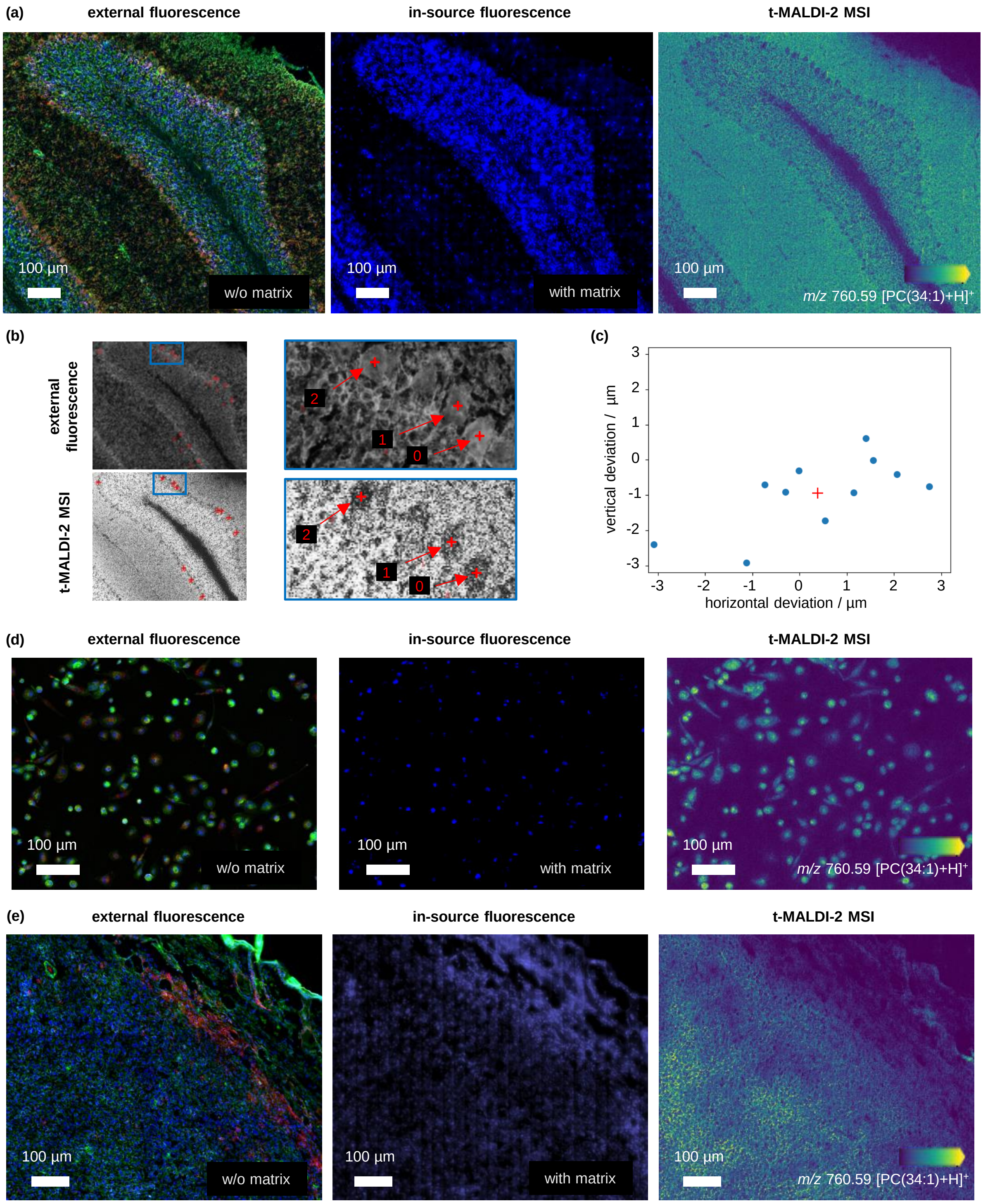

Supplementary Figure 1: Co-registration of external fluorescence image to t-MALDI-2 MSI across the different sample systems

(a) Mouse cerebellum: External fluorescence microscopy (FM) was performed using Hoechst 33342 (nuclei, DAPI channel, blue), CellMask Green Actin Tracking Stain (actin, FITC channel, green), and Alexa Fluor 594 Anti-calbindin antibody (calbindin, Cy3 channel, red). After coating with CHCA matrix by resublimation, an in-source FM image was acquired in the DAPI channel (blue). The t-MALDI-2-MSI data is inherently co-registered with the in-source

FM due to shared optics and stage. In post-processing, the external FM was aligned to the in-source FM and thus to the MSI data.
(b) Eleven Purkinje cell bodies were manually selected in external FM and MSI images to generate marker point pairs. This manual selection is subjective and thus imprecise (see Note S1).
(c) After co-registration, the spatial deviation between marker pairs was measured in x- and y-dimension (blue dots), with the mean deviation indicated (red cross).
(d) THP-1 macrophages: External FM was recorded using Hoechst 33342 (blue), CellMask (green), and pHrodo (phagolysosomes, Cy3 channel, red). After CHCA coating by resublimation, an in-source FM image was acquired in the DAPI channel (blue). MSI is inherently aligned with in-source FM, enabling co-registration of the external FM in post-processing.
(e) Murine 4T1 tumor: External FM was acquired with Hoechst 33342 (blue), CellMask (green), and an anti-Ly6G stain for neutrophils (Cy3 channel, red). After CHCA resublimation, an in-source FM image was recorded in the DAPI channel (blue). The MSI data is inherently aligned with the in-source FM, allowing co-registration of the external FM with both datasets.

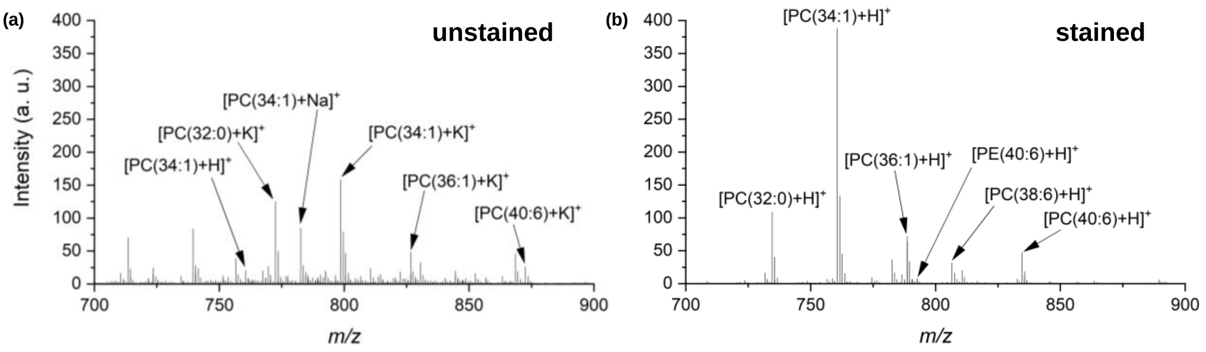

Supplementary Figure 2: Exemplary mass spectra of naïve and processed mouse brain tissue sections
Mass spectra of unstained (a) and stained (b) murine cerebellum sections displaying the *m/z*-range typically occupied by phospholipid signals. Source data is available as a resource data file with this publication.

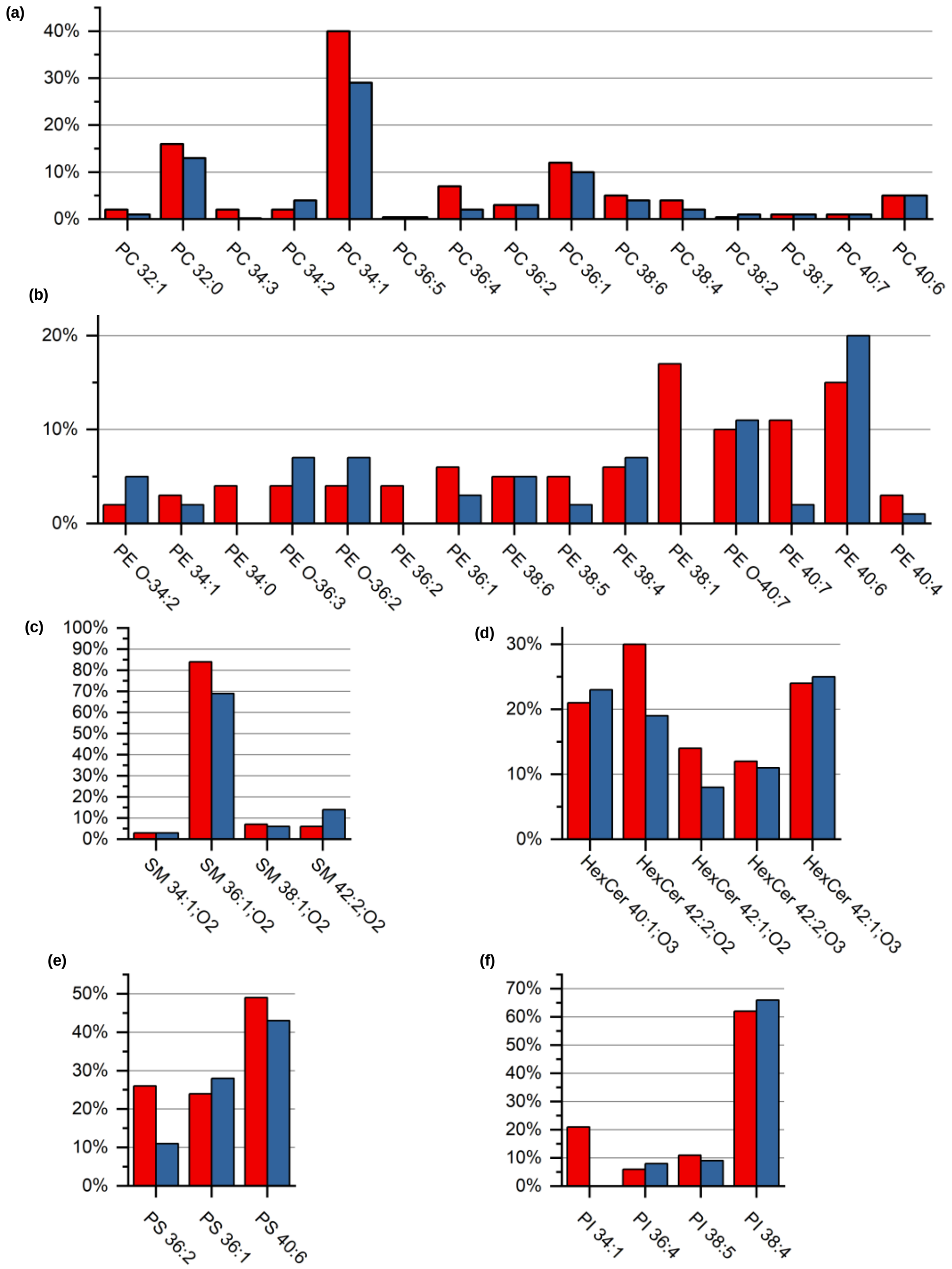

Supplementary Figure 3: Comparison of lipid signal intensities from murine cerebellum acquired with t-MALDI-MSI and reported values from bulk analysis 32

(a) Molar percentage of different types of phosphatidylcholines (PC) in cerebellum derived from Fitzner et al. 32 (blue bars) in comparison with percentages of signal intensities for PCs detected in t-MALDI-2 MSI (red bars) from the whole region depicted in Fig. 1. In both cases values are normalized to the sum of all reported/detected species from this lipid class.
(b) Comparison for phosphatidylethanolamine (PE) species; analogous to (A)
(c) Comparison for sphingomyelin (SM) species; analogous to (A)
(d) Comparison for hexosylceramide (HexCer) species; analogous to (A)
(e) Comparison for phosphatidylserine (PS) species; analogous to (A) (measured in negative ion mode)
(f) Comparison for phosphatidylinositol (PI) species; analogous to (A) (measured in negative ion mode)

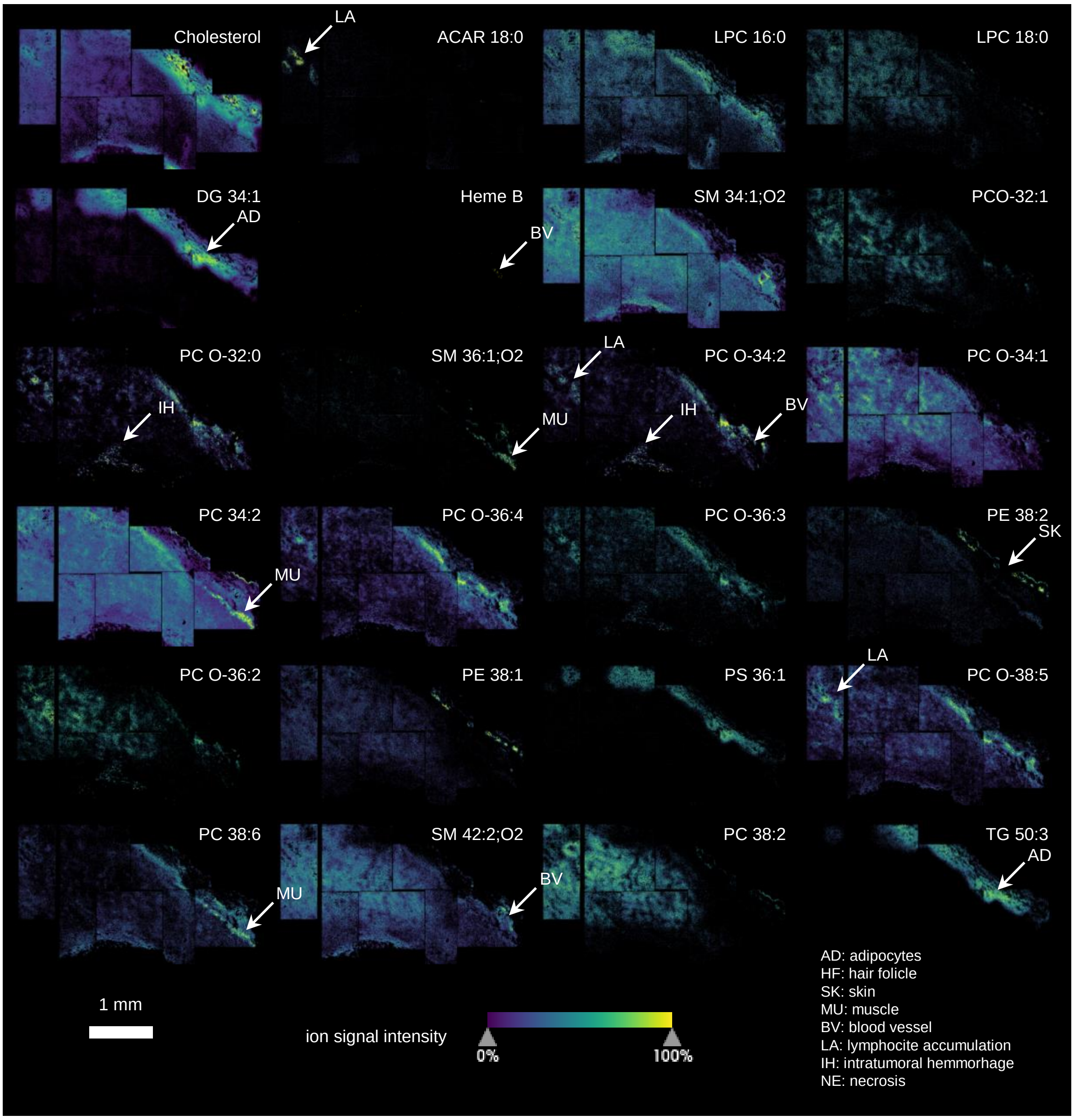

Supplementary Figure 4: Murine 4T1 tumor t-MALDI-2 MSI overview.

Ion signal intensity distributions of different molecules from the measurement in positive ion mode presented in **Fig. 5**. Annotation was performed according to “ion signal annotation” found in the methods.

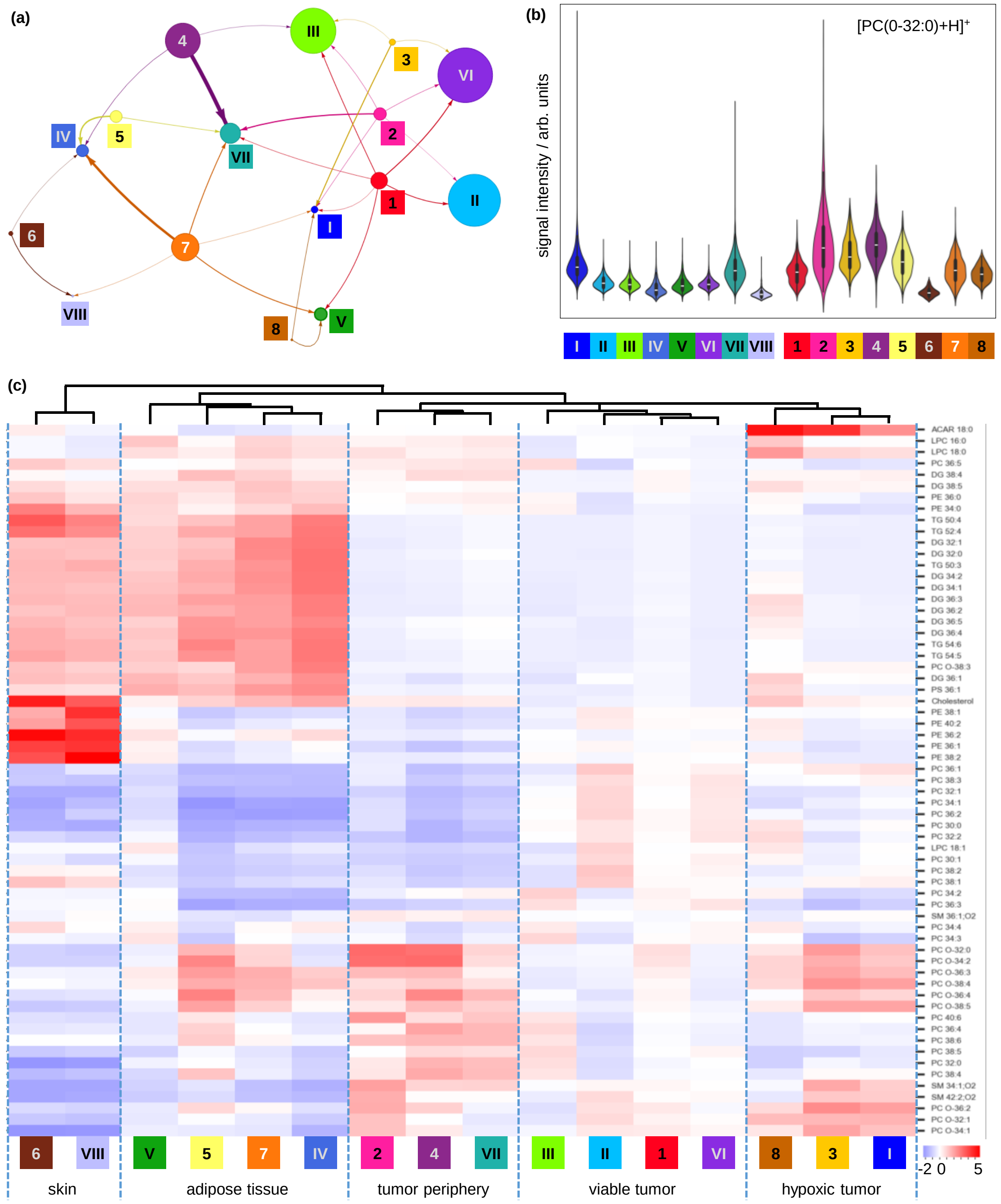

Supplementary Figure 5: Neighborhood analysis and lipid profile of neutrophils in context of their tissue microenvironment

(a) Visualization of the neighborhood analysis for all investigated neutrophils (Fig. 4G). Each circle symbolizes one cluster of cells (Roman numerals) or one cluster of neutrophils (Arabic numerals), respectively. Size of the circle represents the number of underlying cells, for tissue cluster, this number was divided by 10. Arrows and arrow thickness describe the affiliation of neutrophil subtypes to specific tissue clusters.

(b) Violin plots describing the signal intensity distribution for [PC(O-32:0)+H]+ at m/z 720.59 on the cellular level for all tissue clusters and all subtypes of neutrophils, respectively.

(c) The standardized intensities of 63 ion species for all tissue clusters and neutrophils are presented as a heat map to visualize similarities and differences in lipid profiles between the different groups. Annotations of tissue regions are tentative and based on inspection of an adjacent slide stained with H&E as well as clues from FM and MALDI-MSI measurements such as ACAR or DG signal intensities and immune cell agglomerations.

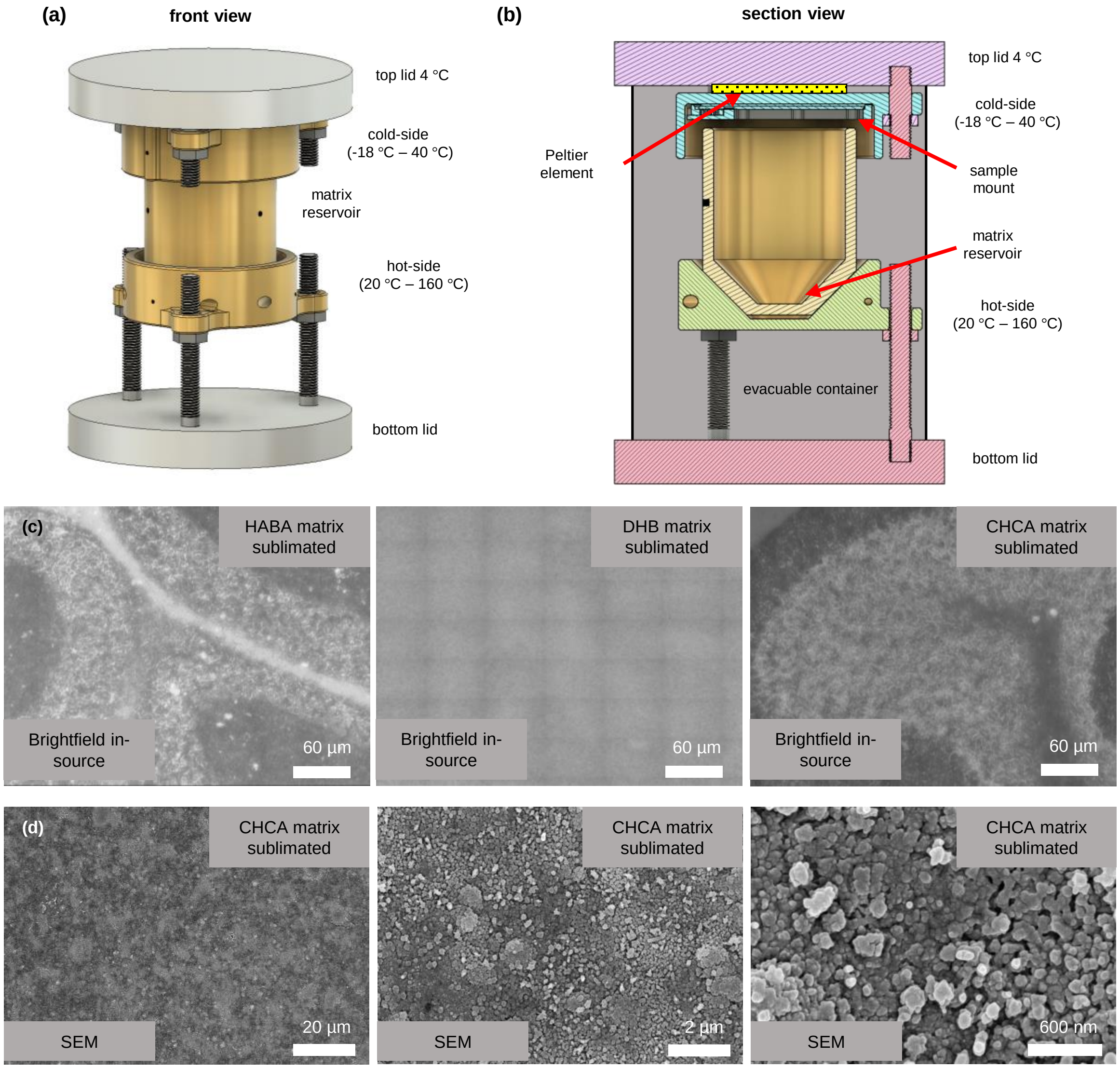

Supplementary Figure 6: Homogenous coating of samples with MALDI-matrix by resublimation.

(a) Schematic of the custom-designed resublimation chamber. The top lid is actively water-cooled to 4 °C and serves as a heatsink for a Peltier element, which can heat up or chill the cold-side from -18 °C to 40 °C. The hot-side is heated by 3 heater cartridges with a total of 300 W heating power. Temperatures of the cold-side and hot-side are monitored using NTC thermistors.

(b) Section view of the custom-designed resublimation chamber. This view of the resublimation chamber reveals the inner details of the setup. The sample can be magnetically clamped to the cold-side. The removable matrix reservoir is contacted to the hot-side using thermally conductive pads. An aluminum tube is mounted between the top and bottom lid to form an evacuable container. Using a turbomolecular pump, the whole setup can be evacuated to 10^{-3} mbar.

(c) In-source BF image of three samples of mouse cerebellum coated with different MALDI matrices by resublimation. The choice of MALDI-matrix heavily influenced the quality of the in-source BF image. Here the specific interplay between matrix and underlying tissue material produces the contrast. With 2-(4-Hydroxyphenylazo)benzoic acid (HABA) and CHCA matrix, the layers of the cerebellum can be easily defined, while resublimation with 2,5-Dihydroxybenzoic acid (DHB) prevents recording of a decent contrast image.

(d) Scanning electron microscopy (SEM) images of a mouse cerebellum coated with CHCA MALDI matrix by resublimation. At three different levels of magnification, the homogenous matrix coating as well as the small matrix crystal size can be observed. A homogeneous coating is crucial to minimize local artifacts in t-MALDI-2 MSI. The matrix crystal size is between 50 nm and 200 nm and therefore is not likely to noticeably limit the t-MALDI-2 MSI lateral resolution.

Supplementary Notes

Supplementary Note 1: Determining the fidelity of co-registration between external FM and MSI

To quantify the accuracy of co-registration between the external FM image and the t-MALDI-2-MSI results, we selected the center of eleven Purkinje cell bodies in the respective images to serve as retrievable pairs of markers (**Supplementary Fig. 1B-C**). The manual placing of each marker at the correct center position of each cell of each individual marker includes individual errors. This results an imprecision in the range of 2-4 µm for each pair. The average across all pairs, however, statistically eliminates the manual placement error and reveals a high fidelity of co-registration with a deviation of less than 1 µm for the x- and y-axis, respectively.

Supplementary Note 2: Annotation of lipids in murine cerebellum and comparison to the literature

Certain fixation and staining steps prior to MALDI-MSI analysis have been described to reduce coverage of the investigated lipidome [2]. These may include chemical crosslinking of amine containing analytes using paraformaldehyde or the depletion of polar lipids during incubation with aqueous staining solutions. To explore the depth of information for lipid analysis available from pre-stained t-MALDI-2-MSI at a pixel size of 1x1 µm², mass spectral information was matched against a full lipidomics profile for murine cerebellum available from the literature [1]. Tentative assignment was based on accurate mass with a tolerance of 3 ppm. This assignment includes the lipid class, the number of carbons and number of double bonds in the fatty acyl chains as well as possible oxidations. It does not differentiate lipid isomers of the same or different lipid classes. On this level of specificity, the conflation of isomeric structures deductible from the literature results in 255 unique *m/z*-values.

Analysis reveals that 82% of the reported molecular lipid content of cerebellum are detected in the t-MALDI-2 measurement. This amounts to 66 *m/z*-values that are detected in t-MALDI-2-MSI producing meaningful intensity distributions in positive and/or negative ion mode measurements (**Table S1**). In particular, this includes lipid classes, such as phosphatidylethanolamine (PE) and phosphatidylserine (PS) that may be affected by prior fixation [2]. Comparison of the detected signal intensities with the molar content of the different lipoforms of each of the detected lipid classes shows a good agreement with the literature (**Supplementary Fig. 3A-F**).

More pronounced deviations from the literature, such as for PC(32:2) that shows a much lower content in MSI may be explained by differences in the employed mouse model; here, the literature is also at variance [3]. Other deviating results such as PE(40:7) and the hexosylceramide HexCer(42:2;O2) may be explained by the selection of the investigated area. Here, specific lipids could be over-or under represented based on the specific tissue type whereas bulk analysis averages over a much larger volume. Some lipid species and lipid classes that occur at very low concentration in brain, such as cardiolipins (CL) or ceramides (Cer) are not detected in the presented t-MALDI-2-MSI data.

Supplementary Tables

Supplementary Table 1: Lipid annotations mouse cerebellum in positive ion mode

m/z-value measured	m/z-value matched	ppm	Name	Formula	Ion	Intensity	rel. intensity in lipid class MSI	rel. intensity in lipid class Fitzner et al.
369.352	369.352	1.1	ST 27:1;O	C27H46O	[M+H-H2O]+	41.836	100%	100%
496.34	496.34	0.4	LPC 16:0	C24H50NO7P	[M+H]+	22.652	100%	45%
577.519	577.519	0.0	DG 34:1	C37H70O5	[M+H-H2O]+	21.782	56%	17%
599.5025	599.503	-1.5	DG 36:4	C39H68O5	[M+H-H2O]+	2.550	7%	8%
605.55	605.55	-0.5	DG 36:1	C39H74O5	[M+H-H2O]+	8.524	22%	14%
627.534	627.535	-1.1	DG 38:4	C41H72O5	[M+H-H2O]+	5.894	15%	33%
702.543	702.543	-0.3	PE O-34:2	C39H76NO7P	[M+H]+	2.516	2%	5%
703.575	703.575	0.3	SM 34:1;O2	C39H79N2O6P	[M+H]+	1.291	3%	3%
718.538	718.538	-0.1	PE 34:1	C39H76NO8P	[M+H]+	3.529	3%	2%
720.5535	720.554	-0.4	PE 34:0	C39H78NO8P	[M+H]+	5.753	4%	0%
728.5585	728.559	-0.5	PE O-36:3	C41H80NO8P	[M+H]+	6.113	4%	7%
730.574	730.575	-0.7	PE O-36:2	C41H80NO7P	[M+H]+	5.910	4%	7%
731.606	731.606	-0.1	SM 36:1;O2	C41H83N2O6P	[M+H]+	36.954	84%	69%
732.554	732.554	0.3	PC 32:1	C40H78NO8P	[M+H]+	17.627	2%	1%
734.5694	734.569	0.0	PC 32:0	C40H80NO8P	[M+H]+	182.308	16%	13%
744.553	744.554	-1.1	PE 36:2	C41H78NO8P	[M+H]+	5.314	4%	0%
746.569	746.569	-0.5	PE 36:1	C41H80NO8P	[M+H]+	7.783	6%	3%
756.5515	756.554	-3.0	PC 34:3	C42H78NO8P	[M+H]+	18.399	2%	0%
758.569	758.569	-0.5	PC 34:2	C42H80NO8P	[M+H]+	22.104	2%	4%
759.637	759.637	-0.5	SM 38:1;O2	C43H87N2O6P	[M+H]+	2.943	7%	6%
760.585	760.585	-0.1	PC 34:1	C42H82NO8P	[M+H]+	441.603	40%	29%
764.5215	764.523	-1.3	PE 38:6	C43H74NO8P	[M+H]+	7.317	5%	5%
766.536	766.538	-2.7	PE 38:5	C43H76NO8P	[M+H]+	7.156	5%	2%
768.553	768.554	-1.0	PE 38:4	C43H78NO8P	[M+H]+	8.507	6%	7%
774.6005	774.601	-0.2582	PE 38:1	C43H84NO8P	[M+H]+	22.933	17%	0%
776.558	776.559	-1.159	PE O-40:7	C45H78NO7P	[M+H]+	13.977	10%	11%
780.552	780.554	-2.3061	PC 36:5	C44H78NO8P	[M+H]+	4.308	0%	0%
782.568	782.569	-1.789	PC 36:4	C44H80NO8P	[M+H]+	73.842	7%	2%
786.6	786.601	-0.8899	PC 36:2	C44H84NO8P	[M+H]+	34.206	3%	3%
788.616	788.616	-0.5072	PC 36:1	C44H86NO8P	[M+H]+	133.613	12%	10%
790.5365	790.538	-2.0239	PE 40:7	C45H76NO8P	[M+H]+	14.707	11%	2%
792.553	792.554	-1.0094	PE 40:6	C45H78NO8P	[M+H]+	20.224	15%	20%
796.584	796.585	-1.3809	PE 40:4	C45H82NO8P	[M+H]+	4.561	3%	1%
800.6604	800.661	-0.7494	HexCer 40:1;O3	C46H89NO9	[M+H]+	1.616	21%	23%
806.569	806.569	-0.4959	PC 38:6	C46H80NO8P	[M+H]+	61.027	5%	4%
810.5995	810.601	-1.4804	PC 38:4	C46H84NO8P	[M+H]+	39.075	4%	2%
810.6815	810.682	-0.2467	HexCer 42:2;O2	C48H91NO8	[M+H]+	2.306	30%	19%
812.695	812.697	-2.9531	HexCer 42:1;O2	C48H93NO8	[M+H]+	1.055	14%	8%
813.6845	813.684	0.1229	SM 42:2;O2	C47H93N2O6P	[M+H]+	2.720	6%	14%
814.631	814.632	-1.2275	PC 38:2	C46H88NO8P	[M+H]+	5.452	0%	1%
816.647	816.648	-0.8572	PC 38:1	C46H90NO8P	[M+H]+	8.948	1%	1%
826.676	826.677	-0.8468	HexCer 42:2;O3	C48H91NO9	[M+H]+	0.915	12%	11%

828.692	828.692	-0.362	HexCer 42:1;O3	C48H93NO9	[M+H]+	1.903	24%	25%
832.584	832.585	-1.3212	PC 40:7	C48H82NO8P	[M+H]+	14.603	1%	1%
834.6005	834.601	-0.2396	PC 40:6	C48H84NO8P	[M+H]+	56.403	5%	5%
836.542	836.544	-1.9126	PS 40:6	C46H78NO10P	[M+H]+	6.345	100%	43%

Supplementary Table 2: Comparison of lipid annotations in stained and naive cerebellum measured in positive ion mode

	stained			naive		
Lipid	$[M+H]^+$	$[M+Na]^+$	$[M+K]^+$	$[M+H]^+$	$[M+Na]^+$	$[M+K]^+$
LPC 16:0	x					x
DG 32:1*				x		
DG 32:0*				x		
DG 34:2*				x		
DG 34:1*	x			x		
DG 34:0*				x		
DG 36:4*	x			x		
DG 36:2*				x		
DG 36:1*	x			x		
DG 38:6*				x		
DG 38:4*	x			x		
PA 32:0						x
PA 34:1					x	x
PA 34:2						x
PA 36:1					x	x
PA 36:2					x	x
PA 36:3						x
PA 36:4				x		x
PA 38:2					x	x
PA 38:3						x
PA 38:4				x		x
PA 38:5				x	x	x
PA 40:5						x
PA 40:6						x
PA 40:7						x
PC 32:0	x			x		x
PC 32:1	x					x
PC 34:1	x			x	x	x
PC 34:2	x			x		x
PC 34:3	x			x		
PC 36:1	x			x	x	x
PC 36:2	x			x	x	x
PC 36:4	x			x	x	x
PC 36:5	x			x		
PC 38:1	x					
PC 38:2	x					
PC 38:4	x			x	x	x
PC 38:6	x			x	x	x
PC 40:6	x			x	x	x
PC 40:7	x			x		
PE 34:0	x			x		
PE 34:1	x			x		
PE 36:0				x	x	
PE 36:1	x			x	x	x
PE 36:2	x			x		x
PE 38:1	x			x		
PE 38:4	x			x	x	x

PE 38:5	x			x		
PE 38:6	x			x		x
PE 40:4	x			x		
PE 40:6	x			x		x
PE 40:7	x			x		
PE O-34:2	x			x		
PE O-36:2	x			x		
PE O-36:3	x			x		
PE O-40:7	x			x		x
PS 40:6	x			x		
SM 34:1;O2	x					
SM 36:1;O2	x				x	x
SM 36:2;O2						x
SM 38:1;O2	x					
SM 42:2;O2	x					
HexCer 40:1;O3	x			x		
HexCer 42:1;O2	x			x		
HexCer 42:1;O3	x			x		
HexCer 42:2;O2	x			x		
HexCer 42:2;O3	x			x		

* detected as $[M+H-H_2O]^+$

Supplementary Table 3: Lipid annotations 4T1-tumor in positive ion mode

m/z-value		ppm	Name	Formula	Ion	Intensity
measured	matched					
369.352	369.3516	1.1	Cholesterol	C27H45	[M+H-H2O]+	72.293
428.374	428.3734	1.4	ACAR 18:0	C25H50NO4	[M+H]+	6.170
496.34	496.3398	0.4	LPC 16:0	C24H50NO7P	[M+H]+	20.172
522.356	522.3554	1.1	LPC 18:1	C26H52NO7P	[M+H]+	9.851
524.3715	524.3711	0.8	LPC 18:0	C26H54NO7P	[M+H]+	10.990
549.488	549.4877	0.5	DG 32:1	C35H66O5	[M+H-H2O]+	7.819
551.5036	551.5034	0.4	DG 32:0	C35H68O5	[M+H-H2O]+	14.607
575.503	575.5034	0.7	DG 34:2	C37H68O5	[M+H-H2O]+	12.517
577.519	577.519	0.0	DG 34:1	C37H70O5	[M+H-H2O]+	20.028
597.487	597.4877	1.2	DG 36:5	C39H66O5	[M+H-H2O]+	2.173
599.503	599.5034	0.7	DG 36:4	C39H68O5	[M+H-H2O]+	6.174
601.5185	601.519	0.8	DG 36:3	C39H70O5	[M+H-H2O]+	8.774
603.5344	603.5347	0.5	DG 36:2	C39H72O5	[M+H-H2O]+	13.483
605.55	605.5503	0.5	DG 36:1	C39H74O5	[M+H-H2O]+	7.045
625.5184	625.519	1.0	DG 38:5	C41H70O5	[M+H-H2O]+	2.929
627.5343	627.5347	0.6	DG 38:4	C41H72O5	[M+H-H2O]+	5.852
703.575	703.5748	0.3	SM 34:1;O2	C39H79N2O6P	[M+H]+	62.825
704.5225	704.5225	0.0	PC 30:1	C38H74NO8P	[M+H]+	5.182
706.538	706.5381	0.1	PC 30:0	C38H76NO8P	[M+H]+	36.007
718.5745	718.5745	0.0	PC O-32:1	C40H80NO7P	[M+H]+	8.277
720.5535	720.5538	0.4	PE 34:0	C39H78NO8P	[M+H]+	8.659
720.59	720.5902	0.3	PC O-32:0	C40H82NO7P	[M+H]+	16.148
730.538	730.5381	0.1	PC 32:2	C40H76NO8P	[M+H]+	10.551
731.606	731.6061	0.1	SM 36:1;O2	C41H83N2O6P	[M+H]+	4.116
732.554	732.5538	0.3	PC 32:1	C40H78NO8P	[M+H]+	78.153
734.569	734.5694	0.5	PC 32:0	C40H80NO8P	[M+H]+	160.317
744.5535	744.5538	0.4	PE 36:2	C41H78NO8P	[M+H]+	4.134
744.59	744.5902	0.3	PC O-34:2	C42H82NO7P	[M+H]+	11.722
746.5695	746.5694	0.1	PE 36:1	C41H80NO8P	[M+H]+	9.331
746.606	746.6058	0.3	PC O-34:1	C42H84NO7P	[M+H]+	34.910
748.5845	748.5851	0.8	PE 36:0	C41H82NO8P	[M+H]+	8.611
754.5375	754.5381	0.8	PC 34:4	C42H76NO8P	[M+H]+	4.032
756.553	756.5538	1.1	PC 34:3	C42H78NO8P	[M+H]+	18.459
758.569	758.5694	0.5	PC 34:2	C42H80NO8P	[M+H]+	133.665
760.585	760.5851	0.1	PC 34:1	C42H82NO8P	[M+H]+	250.313
768.5893	768.5902	1.2	PC O-36:4	C44H82NO7P	[M+H]+	18.033
770.6045	770.6058	1.7	PC O-36:3	C44H84NO7P	[M+H]+	8.439
772.5844	772.5851	0.9	PE 38:2	C43H82NO8P	[M+H]+	7.695
772.6205	772.6215	1.3	PC O-36:2	C44H86NO7P	[M+H]+	7.037
774.6	774.6007	0.9	PE 38:1	C43H84NO8P	[M+H]+	10.802
780.552	780.5538	2.3	PC 36:5	C44H78NO8P	[M+H]+	9.924
782.568	782.5694	1.8	PC 36:4	C44H80NO8P	[M+H]+	72.343
784.584	784.5851	1.4	PC 36:3	C44H82NO8P	[M+H]+	67.002

786.6	786.6007	0.9	PC 36:2	C44H84NO8P	[M+H]+	165.845
788.615	788.6164	1.8	PC 36:1	C44H86NO8P	[M+H]+	88.551
790.559	790.5593	0.4	PS 36:1	C42H80NO10P	[M+H]+	4.668
794.6046	794.6058	1.5	PC O-38:5	C46H84NO7P	[M+H]+	17.134
796.62	796.6215	1.9	PC O-38:4	C46H86NO7P	[M+H]+	10.837
798.635	798.6371	2.6	PC O-38:3	C46H88NO7P	[M+H]+	4.026
800.615	800.6164	1.7	PE 40:2	C45H86NO8P	[M+H]+	5.439
806.568	806.5694	1.7	PC 38:6	C46H80NO8P	[M+H]+	12.241
808.584	808.5851	1.4	PC 38:5	C46H82NO8P	[M+H]+	29.400
810.6	810.6007	0.9	PC 38:4	C46H84NO8P	[M+H]+	70.487
812.614	812.6164	3.0	PC 38:3	C46H86NO8P	[M+H]+	25.856
813.6835	813.6844	1.1	SM 42:2;O2	C47H93N2O6P	[M+H]+	23.151
814.6305	814.632	1.8	PC 38:2	C46H88NO8P	[M+H]+	13.575
816.6455	816.6477	2.7	PC 38:1	C46H90NO8P	[M+H]+	5.026
827.711	827.7123	1.6	TG 50:4	C53H94O6	[M+H]+	2.731
829.727	829.728	1.2	TG 50:3	C53H96O6	[M+H]+	4.031
834.599	834.6007	2.0	PC 40:6	C48H84NO8P	[M+H]+	7.698
855.7425	855.7436	1.3	TG 52:4	C55H98O6	[M+H]+	6.736
879.742	879.7436	1.8	TG 54:6	C57H98O6	[M+H]+	2.774
881.7576	881.7593	1.9	TG 54:5	C57H100O6	[M+H]+	3.935

Supplementary Table 4: Table of resources

REAGENT or RESOURCE	SOURCE	IDENTIFIER
Antibodies		
Anti-Calbindin antibody	Abcam	Cat# ab229915; RRID:AB_3086776
Goat Anti-Rabbit IgG H&L (Alexa Fluor® 594)	Abcam	Cat# ab150080; RRID:AB_2650602
Alexa Fluor® 594 Anti-Ly6g antibody [EPR22909-135]	Abcam	Cat# ab307167
DcTRAIL-R1 (TNFRH1) Antibody, anti-mouse (APC-Vio® 770)	Miltenyi Biotec	Cat# 130-110-873; RRID:AB_2651535
CD45 Antibody, anti-mouse, APC, REAfinity	Miltenyi Biotec	Cat# 130-110-798; RRID:AB_2658220
Chemicals, peptides, and recombinant proteins		
CellMask™ Green Actin Tracking Stain	Thermo Fisher	Cat# A57243
Hoechst 33342	Sigma-Aldrich	Cat# 14533
pHrodo™ Red E. coli BioParticles™ Conjugate for Phagocytosis	Thermo Fisher	Cat# P35361
Ammonium acetate	Sigma-Aldrich	Cat# A1542
Gibco™ PBS, pH 7.4	Thermo Fisher	Cat# 12579099
Albumin bovine Fraction V, pH 7.0 (BSA)	Serva	Cat# 11930.03
RPMI 1640 Medium	Lonza	Cat# BE15-398D
L-glutamine	Lonza	Cat# BE17-605F
Fetal bovine serum	Sigma-Aldrich	Cat# S0615
Sodium pyruvate	Sigma-Aldrich	Cat# S8636
phorbol 12-myristate 13-acetate (PMA)	Sigma-Aldrich	Cat# P1585
Formaldehyde 30 %, low-methanol	Roth	Cat# 4235.1
Mayer’s hemalum solution	Sigma-Aldrich	Cat# 109249
Eosin Y-solution 0.5% aqueous	Sigma-Aldrich	Cat# 109844
α-Cyano-4-hydroxycinnamic acid (CHCA)	Sigma-Aldrich	Cat# 70990
2-(4-Hydroxyphenylazo)benzoic acid (HABA)	Sigma-Aldrich	Cat# 54793
2,5-Dihydroxybenzoic acid (DHB)	Sigma-Aldrich	Cat# 85707
Epredia™ M-1 Embedding Matrix	Thermo Fisher	Cat# 10056778
DMEM, high glucose, GlutaMAX™	Thermo Fisher	Cat# 61965026
Deposited data		
MALDI-MSI raw and process data	This paper	will be available at the time of publication
Fluorescence and brightfield microscopy data	This paper	will be available at the time of publication
Cell masks and mass spectra	This paper	will be available at the time of publication
Experimental models: Cell lines		
Human: THP-1 cells	DSMZ	ACC-16
Experimental models: Organisms/strains		
Mice	Charles River Laboratories, Sulzfeld, Germany	Female BALB/c
Software and algorithms		

Python version 3.8	Python Software Foundation	https://www.python.org
ImageJ	Rasband [4]	https://imagej.net/ij/
XC-SDK 2018	Sony	https://www.image-sensing-solutions.eu/XCG-CG160.html
SCiLS Lab MVS, Version 2024b Pro	Bruker Daltonics	https://www.bruker.com/en/products-and-solutions/mass-spectrometry/ms-software/scils-lab.html
flexImaging 7.5 R&D Prototype	Bruker Daltonics	
timsControl 6.0.0 alpha	Bruker Daltonics	
OlyVIA 4.1	Evident	https://www.olympus-lifescience.com/de/discovery/image-sharing-made-easy-meet-olyvia/
FISCAS	Schwenzfeier et al. [5]	https://github.com/BioMedMS/fiscas
SimpleITK 2.3.1	NumFOCUS [6,7]	https://simpleitk.org
DeepCell Mesmer 0.12.9	Van Valen Lab [8]	https://www.deepcell.org/
CellProfiler 4.2.1	Cimini Lab [9]	https://cellprofiler.org/
LipostarMSI 2.0.1	Molecular Horizon [10]	https://www.molhorizon.it/software/lipostar/
opencv-Python-headless 4.6.0.66	OpenCV Team	https://pypi.org/project/opencv-python-headless/
imageio 2.22.4	Almar Klein	https://pypi.org/project/imageio/
jupyterlab 4.2.3	Project Jupyter	https://jupyter.org/
matplotlib 3.6.3	Matplotlib [11]	https://matplotlib.org/
numba 0.56.4	Numba	https://numba.pydata.org/
numpy 1.23.4	Numpy [12]	https://numpy.org/
pandas 1.5.1	Pandas [13]	https://pandas.pydata.org/
scikit-image 0.19.3	Scikit-Image [14]	https://scikit-image.org/
scipy 1.9.3	SciPy [15]	https://scipy.org/
seaborn 0.13.2	Seaborn [16]	https://seaborn.pydata.org/
tqdm 4.64.1	Tqdm [17]	https://pypi.org/project/tqdm/

umap-learn 0.5.6	UMAP [18]	https://pypi.org/project/umap-learn/
Fusion 2.0.20256	Autodesk	www.autodesk.com
Other		
Millicell EZ Slide 8-well chamber slides	Sigma-Aldrich	Cat# PEZGS0816
t-MALDI-2 mass spectrometer	Bruker	modified timsTOF fleX MALDI-2; this paper
Orbitrap mass spectrometer	Thermo Fisher	Q-Exactive Plus
Resublimation chamber	This paper	self-built
Cryostat	Leica Biosystems	Cat# CM 3050 S
Super PAP Pen Liquid Blocker	Science Services	Cat# N71310-N
MALDI IntelliSlides	Bruker Daltonics	Cat# 1868957
SuperFrost slides	Thermo Fisher	Cat# 17284884
Piezo sample stage	SmarAct	Custom Design
Objective M Plan Apo NUV HR 50X	Mitutoyo	Cat# 378-888-6
Tube lens 100 mm	Thorlabs	Cat# TTL100-A
Digital Camera	Sony	XCG-CG160
Dichroic Mirror HR355nm/45° Typ1 on FS-LO-V Ø25x6.35mm	Laseroptik	Cat# L-07246
Green LED	Thorlabs	Cat# LED528EHP
Filter Cube	Thorlabs	Cat# DFM2/M
Filter Cube Insert	Thorlabs	Cat# DFM2T1
DAPI LED	Thorlabs	Cat# M385FP1
FITC LED	Thorlabs	Cat# M455F3
mCherry LED	Thorlabs	Cat# MINTF4
DAPI Filter Set	Thorlabs	Cat# MDF-BFP
FITC Filter Set	Thorlabs	Cat# MDF-FITC
mCherry Filter Set	Thorlabs	Cat# MDF-MCHC
1951 USAF Resolution Test Targets, 3" x 1"	Thorlabs	Cat# R3L1S4P
VS200 Research Microscopy Slide Scanner	Olympus/Evident	VS200
Digital Delay Pulse Generator	Quantum Composers	9200 Sapphire Series

Supplementary table 5: Light microscopy reporting table

Hardware

**Instrument and Camera**	*VS200 Research Slide Scanner (Olympus/Evident) with ORCA-Fusion #2 (Hamamatsu)*
**Resolution**	*50xMPLAPO NA=0.95*
**Filters and Light Sources**	*X-Cite Novem, DAPI, FITC, KSS TxRed, KSS CY5, KSS CY7*

Quality Control

Is this the first report of the instrument? If yes, response to the below points is required and providing validation data is encouraged.

Yes	x
No	

For all instruments, confirm that the following quality control processes were run:

n/a	Confirmed		
x		Field homogeneity is assessed.	
x		Illumination power stability is tested.	
x		Stage drift is assessed.	
x		Camera/detector noise is measured.	

Methodology

Sample preparation and imaging

Stained specimens were air-dried on slides and imaged without mounting medium or coverslip using a dry (air) objective. Imaging was performed at ambient room temperature (~22–24 °C; no active temperature control).

Fluorophores

CellMask™ Green Actin Tracking Stain	Thermo Fisher	Cat# A57243
Hoechst 33342	Sigma-Aldrich	Cat# 14533
LipidSpot™ 610	Biotium	Cat# 70069-T
Anti-Calbindin antibody	Abcam	Cat# ab229915; RRID:AB_3086776
Goat Anti-Rabbit IgG H&L (Alexa Fluor® 594)	Abcam	Cat# ab150080; RRID:AB_2650602
Alexa Fluor® 594 Anti-Ly6g antibody [EPR22909-135]	Abcam	Cat# ab307167
DcTRAIL-R1 (TNFRH1) Antibody, anti-mouse (APC-Vio® 770)	Miltenyi Biotec	Cat# 130-110-873; RRID:AB_2651535
CD45 Antibody, anti-mouse, APC, REAfinity	Miltenyi Biotec	Cat# 130-110-798; RRID:AB_2658220

Acquisition

**Software**	*OLYMPUS VS200 ASW 4.1.1 (Build 29408)*

**Enhancements**	*None*
**Imaging parameters**	*Global max exposure limited to 500 ms. For each channel, auto-exposure was run on a bright ROI; the resulting exposure was then applied uniformly to the whole slide. Typical exposures: DAPI ~10 ms; FITC ~200 ms; longer-wavelength channels 400–500 ms.*
**Size**	*130 nm/pixel*

Image Processing

**Software**	*OLYMPUS OlyVIA 3.4.1 (Build 26606)*
**Workflow**	Post-acquisition, only linear display histogram (min/max) adjustments were made per channel to enhance cell visibility; raw pixel data otherwise unprocessed. No deconvolution, gamma change, filtering, thresholding, projection, or 3D rendering applied.

References Supplementary Information

1. Fitzner, D. *et al.* Cell-Type- and Brain-Region-Resolved Mouse Brain Lipidome. *Cell Rep.* **32**, 108132 (2020).
2. Vos, D. R. N., Bowman, A. P., Heeren, R. M. A., Balluff, B. & Ellis, S. R. Class-specific depletion of lipid ion signals in tissues upon formalin fixation. *Int. J. Mass Spectrom.* **446**, 116212 (2019).
3. Vandenbosch, M. *et al.* Toward Omics-Scale Quantitative Mass Spectrometry Imaging of Lipids in Brain Tissue Using a Multiclass Internal Standard Mixture. *Anal. Chem.* **95**, 18719–18730 (2023).
4. Schneider, C. A., Rasband, W. S. & Eliceiri, K. W. NIH Image to ImageJ: 25 years of image analysis. *Nat Methods* **9**, 671–675 (2012).
5. Schwenzfeier, J., Weischer, S., Bessler, S. & Soltwisch, J. Introducing FISCAS, a Tool for the Effective Generation of Single Cell MALDI-MSI Data. *J. Am. Soc. Mass Spectrom.* **35**, 2950–2959 (2024).
6. Yaniv, Z., Lowekamp, B. C., Johnson, H. J. & Beare, R. SimpleITK Image-Analysis Notebooks: a Collaborative Environment for Education and Reproducible Research. *J Digit Imaging* **31**, 290–303 (2018).
7. Lowekamp, B. C., Chen, D. T., Ibanez, L. & Blezek, D. The Design of SimpleITK. *Front. Neuroinform.* **7**, (2013).
8. Greenwald, N. F. *et al.* Whole-cell segmentation of tissue images with human-level performance using large-scale data annotation and deep learning. *Nat. Biotechnol.* **40**, 555–565 (2022).
9. Stirling, D. R. *et al.* CellProfiler 4: improvements in speed, utility and usability. *BMC Bioinf.* **22**, 433 (2021).
10. Tortorella, S. *et al.* LipostarMSI: Comprehensive, Vendor-Neutral Software for Visualization, Data Analysis, and Automated Molecular Identification in Mass Spectrometry Imaging. *J. Am. Soc. Mass Spectrom.* **31**, 155–163 (2020).
11. Hunter, J. D. Matplotlib: A 2D Graphics Environment. *Computing in Science & Engineering* **9**, 90–95 (2007).
12. Harris, C. R. *et al.* Array programming with NumPy. *Nature* **585**, 357–362 (2020).
13. McKinney, W. Data Structures for Statistical Computing in Python. *scipy* (2010) doi:10.25080/Majora-92bf1922-00a.
14. Walt, S. van der *et al.* scikit-image: image processing in Python. *PeerJ* **2**, e453 (2014).
15. Virtanen, P. *et al.* SciPy 1.0: fundamental algorithms for scientific computing in Python. *Nat Methods* **17**, 261–272 (2020).
16. Waskom, M. L. seaborn: statistical data visualization. *Journal of Open Source Software* **6**, 3021 (2021).
17. Costa-Luis, C. da *et al.* tqdm: A fast, Extensible Progress Bar for Python and CLI. Zenodo https://doi.org/10.5281/zenodo.14231923 (2024).
18. Healy, J. & McInnes, L. Uniform manifold approximation and projection. *Nat Rev Methods Primers* **4**, 1–15 (2024).
