# Supplementary material for: Spatial biology using single-cell mass spectrometry imaging and integrated microscopy
Source: Nat Commun. 2025 Oct 15;16:9129. doi: 10.1038/s41467-025-64603-8 (PMC12528677; doi:10.1038/s41467-025-64603-8)
Supplement: Supplementary file 2 — Reporting Summary [file 41467_2025_64603_MOESM2_ESM.pdf]

nature portfolio

Corresponding author(s): Jens Soltwisch

Last updated by author(s): Sep 8, 2025

Reporting Summary

Nature Portfolio wishes to improve the reproducibility of the work that we publish. This form provides structure for consistency and transparency in reporting. For further information on Nature Portfolio policies, see our Editorial Policies and the Editorial Policy Checklist.

Statistics

For all statistical analyses, confirm that the following items are present in the figure legend, table legend, main text, or Methods section.

n/a	Confirmed	
☐	☒	The exact sample size (*n*) for each experimental group/condition, given as a discrete number and unit of measurement
☐	☒	A statement on whether measurements were taken from distinct samples or whether the same sample was measured repeatedly
☐	☒	The statistical test(s) used AND whether they are one- or two-sided<br>*Only common tests should be described solely by name; describe more complex techniques in the Methods section.*
☐	☒	A description of all covariates tested
☐	☒	A description of any assumptions or corrections, such as tests of normality and adjustment for multiple comparisons
☐	☒	A full description of the statistical parameters including central tendency (e.g. means) or other basic estimates (e.g. regression coefficient) AND variation (e.g. standard deviation) or associated estimates of uncertainty (e.g. confidence intervals)
☒	☐	For null hypothesis testing, the test statistic (e.g. *F*, *t*, *r*) with confidence intervals, effect sizes, degrees of freedom and *P* value noted<br>*Give P values as exact values whenever suitable.*
☒	☐	For Bayesian analysis, information on the choice of priors and Markov chain Monte Carlo settings
☐	☒	For hierarchical and complex designs, identification of the appropriate level for tests and full reporting of outcomes
☐	☒	Estimates of effect sizes (e.g. Cohen's *d*, Pearson's *r*), indicating how they were calculated

Our web collection on statistics for biologists contains articles on many of the points above.

Software and code

Policy information about availability of computer code

Data collection	Python version 3.8 Python Software Foundation https://www.python.org<br>XC-SDK 2018 Sony https://www.image-sensing-solutions.eu/XCG-CG160.html<br>flexImaging 7.5 R&D Prototype Bruker Daltonics<br>timsControl 6.0.0 alpha Bruker Daltonics
Data analysis	Python version 3.8 Python Software Foundation https://www.python.org<br>ImageJ Rasband 62 https://imagej.net/ij/<br>SCiLS Lab MVS, Version 2024b Pro Bruker Daltonics https://www.bruker.com/en/products-and-solutions/mass-spectrometry/ms-software/scils-lab.html<br>OlyVIA 4.1 Evident https://www.olympus-lifescience.com/de/discovery/image-sharing-made-easy-meet-olyvia/<br>FISCAS Schwenzfeier et al. 48 https://github.com/BioMedMS/fiscas<br>SimpleITK 2.3.1 NumFOCUS 63,64 https://simpleitk.org<br>DeepCell Mesmer 0.12.9 Van Valen Lab 49 https://www.deepcell.org/<br>CellProfiler 4.2.1 Cimini Lab 50 https://cellprofiler.org/<br>LipostarMSI 2.0.1 Molecular Horizon 65 https://www.molhorizon.it/software/lipostar/<br>opencv-Python-headless 4.6.0.66 OpenCV Team https://pypi.org/project/opencv-python-headless/<br>imageio 2.22.4 Almar Klein https://pypi.org/project/imageio/<br>jupyterlab 4.2.3 Project Jupyter https://jupyter.org/<br>matplotlib 3.6.3 Matplotlib 66 https://matplotlib.org/<br>numba 0.56.4 Numba https://numba.pydata.org/

numpy 1.23.4 Numpy 67 https://numpy.org/
pandas 1.5.1 Pandas 68 https://pandas.pydata.org/
scikit-image 0.19.3 Scikit-Image 69 https://scikit-image.org/
scipy 1.9.3 SciPy 70 https://scipy.org/
seaborn 0.13.2 Seaborn 71 https://seaborn.pydata.org/
tqdm 4.64.1 Tqdm 72 https://pypi.org/project/tqdm/
umap-learn 0.5.6 UMAP 73 https://pypi.org/project/umap-learn/

For manuscripts utilizing custom algorithms or software that are central to the research but not yet described in published literature, software must be made available to editors and reviewers. We strongly encourage code deposition in a community repository (e.g. GitHub). See the Nature Portfolio guidelines for submitting code & software for further information.

Data

Policy information about availability of data

All manuscripts must include a data availability statement. This statement should provide the following information, where applicable:

- Accession codes, unique identifiers, or web links for publicly available datasets
- A description of any restrictions on data availability
- For clinical datasets or third party data, please ensure that the statement adheres to our policy

MALDI MSI data, fluorescence and brightfield microscopy images as well as processed data have been deposited at OMERO and are publicly available including an interactive data viewer at https://doi.org/10.57860/min_prj_000012 . Any additional information required to open or reanalyze the data reported in this paper is available from the lead contact upon request. Source data are provided with this paper.

Research involving human participants, their data, or biological material

Policy information about studies with human participants or human data. See also policy information about sex, gender (identity/presentation), and sexual orientation and race, ethnicity and racism.

Reporting on sex and gender	No research involving human participants.
Reporting on race, ethnicity, or other socially relevant groupings	No research involving human participants.
Population characteristics	No research involving human participants.
Recruitment	No research involving human participants.
Ethics oversight	No research involving human participants.

Note that full information on the approval of the study protocol must also be provided in the manuscript.

Field-specific reporting

Please select the one below that is the best fit for your research. If you are not sure, read the appropriate sections before making your selection.

[x] Life sciences [ ] Behavioural & social sciences [ ] Ecological, evolutionary & environmental sciences

For a reference copy of the document with all sections, see nature.com/documents/nr-reporting-summary-flat.pdf

Life sciences study design

All studies must disclose on these points even when the disclosure is negative.

Sample size	The included data for the presented proof-of-concept studies are each based on a biological sample size of one.
Data exclusions	No data was excluded from the study
Replication	Studies on mouse brain and cell culture where repeated at least once and produced comparable results. Measurements for the study of neutrophils in tumor were repeated and produced comparable data. Full data analysis was not repeated for the tumor study
Randomization	No randomization was done, because of the sample size of one.
Blinding	Blinding between samples was not releveant to the study because of the limited sample size. Clustering of individual cells was based on MS and fluorescence data alone without knowledge on their location within the sample.

Reporting for specific materials, systems and methods

We require information from authors about some types of materials, experimental systems and methods used in many studies. Here, indicate whether each material, system or method listed is relevant to your study. If you are not sure if a list item applies to your research, read the appropriate section before selecting a response.

Materials & experimental systems

n/a	Involved in the study
☐	☒ Antibodies
☐	☒ Eukaryotic cell lines
☒	☐ Palaeontology and archaeology
☐	☒ Animals and other organisms
☒	☐ Clinical data
☒	☐ Dual use research of concern
☒	☐ Plants

Methods

n/a	Involved in the study
☒	☐ ChIP-seq
☒	☐ Flow cytometry
☒	☐ MRI-based neuroimaging

Antibodies

Antibodies used	Anti-Calbindin antibody Abcam Cat# ab229915; RRID:AB_3086776<br>Goat Anti-Rabbit IgG H&L (Alexa Fluor® 594) Abcam Cat# ab150080; RRID:AB_2650602<br>Alexa Fluor® 594 Anti-Ly6g antibody [EPR22909-135] Abcam Cat# ab307167<br>DcTRAIL-R1 (TNFRH1) Antibody, anti-mouse (APC-Vio® 770) Miltenyi Biotec Cat# 130-110-873; RRID:AB_2651535<br>CD45 Antibody, anti-mouse, APC, REAfinity Miltenyi Biotec Cat# 130-110-798; RRID:AB_2658220
Validation	All antibodies were marked as tested (Abcam) or "Satisfaction quaranteed" (Miltenyi) upon purchase

Eukaryotic cell lines

Policy information about cell lines and Sex and Gender in Research

Cell line source(s)	Human: THP-1 cells DSMZ ACC-16
Authentication	The cell line was purchased directly from the DSMZ and not authenticated after arival.
Mycoplasma contamination	The cell line was not tested for mycoplasma contamination.
Commonly misidentified lines (See ICLAC register)	no commonly misidentified lines were used.

Animals and other research organisms

Policy information about studies involving animals; ARRIVE guidelines recommended for reporting animal research, and Sex and Gender in Research

Laboratory animals	Female BALB/c mice (Charles River Laboratories)
Wild animals	No wild animals were used.
Reporting on sex	The breast cancer model only applies to female mice. The brain study only concerns structures in the brain that are independent of the sex.
Field-collected samples	No field collected samples were used.
Ethics oversight	North Rhine-Westphalia State Agency for Nature, Environment and Consumer Protection, LANUV, Protocol No. 81-02.04.2018.A010

Note that full information on the approval of the study protocol must also be provided in the manuscript.

Plants

Seed stocks	No plants were used.
Novel plant genotypes	No plants were used.
Authentication	No plants were used.
